# Revision Surgery for a Foot With Loss of Correction After Modified Lapidus Procedure: A Case Report

**DOI:** 10.7759/cureus.80372

**Published:** 2025-03-10

**Authors:** Hikari Okuda, Tadashi Kimura, Mitsuru Saito, Makoto Kubota

**Affiliations:** 1 Department of Orthopaedic Surgery, Jikei University School of Medicine, Tokyo, JPN

**Keywords:** corrective loss, dorsiflexion of the first metatarsal, hallux valgus, lapidus procedure, medial longitudinal arch, revision surgery

## Abstract

We report a case of revision surgery for a foot with loss of correction after a modified Lapidus procedure. The patient was a 68-year-old woman, with advanced hallux valgus in both feet.

First, a modified Lapidus procedure and Weil osteotomy were performed on the second ray in the left foot, with a good postoperative course. Two months later, she underwent a modified Lapidus procedure on the right foot to correct severe deformity, with a preoperative hallux valgus angle of 55° to 9° and a first-second intermetatarsal (M1M2) angle of 26° to 8°. However, correction was lost early after the surgery. Plain radiographs of the right foot, obtained two months after surgery, showed a hallux valgus angle of 24° and an M1M2 angle of 14° with no bone union. Considering that the first metatarsal was dorsiflexed and adducted around the first tarsometatarsal joint, revision surgery with additional screws and an iliac bone graft was performed, which resulted in good correction with no recurrence. Dorsiflexion of the first metatarsal was thought to be the main reason for the recurrence of hallux valgus in this case. The likely reason was that when the hallux valgus recurred, dorsiflexion of the first metatarsal was greater than the opening M1M2 angle, with less rotation of the first metatarsal head.

We believe that alignment correction in the sagittal plane is one of the most important aspects of the correction of hallux valgus.

## Introduction

In general, distal osteotomy is used for mild deformity, and proximal osteotomy, metaphyseal osteotomy, and the first tarsometatarsal (TMT) joint fixation (modified Lapidus procedure) are used for moderate to severe deformity. Various surgical methods have been proposed, but there is no unified technique because of frequent recurrence [1].

Correction of hallux valgus deformity has traditionally focused on correction of the first-second intermetatarsal (M1M2) angle. It has also been considered necessary to rotate the first ray and reduce the sesamoid bone [2]. In recent years, reproduction of the arch of the foot - that is, realignment in the sagittal plane - is also considered to be important in cases with advanced deformity [2,3].

We believe that proximal osteotomy is useful because it allows us to reproduce the arch in three dimensions rather than just correcting it in two dimensions. Proximal osteotomy and a modified Lapidus procedure are effective in recreating the medial longitudinal arch with hallux valgus. In proximal osteotomies with three-dimensional correction, metatarsus latus and a flat foot can be eliminated by recreating the arch, and postoperative reduction of hypermobility of the first ray can be seen [4].

However, a large correction angle results in an unnatural postoperative foot. When the rotation is corrected, the bone joint surfaces are difficult to align, and the recurrence of deformity is common [5]. In contrast, a modified Lapidus procedure can also directly address hypermobility of the first TMT joint [2,3]. This procedure corrects the deformity close to its center [6], and bone union is achieved with cancellous bone. Therefore, the foot morphology is good, and bone union is easily achieved, even after significant angular correction.

For patients with severe hallux valgus, significant hypermobility of the first TMT joint, and bone fragility, such as the elderly, osteoporosis, and diabetes mellitus, the modified Lapidus procedure has recently been performed, and its efficacy has been reevaluated, which we also perform [2,3].

We describe a case in which loss of correction after a modified Lapidus procedure required revision surgery with a focus on alignment in the sagittal plane and achieved good correction.

## Case presentation

The patient was a 68-year-old woman who was being treated for rheumatoid arthritis with immunomodulating agents. She developed bilateral hallux valgus at around 40 years of age, and the deformity gradually worsened. She was referred to our hospital because of worsening pain on walking at around 60 years of age.

**Figure 1:**
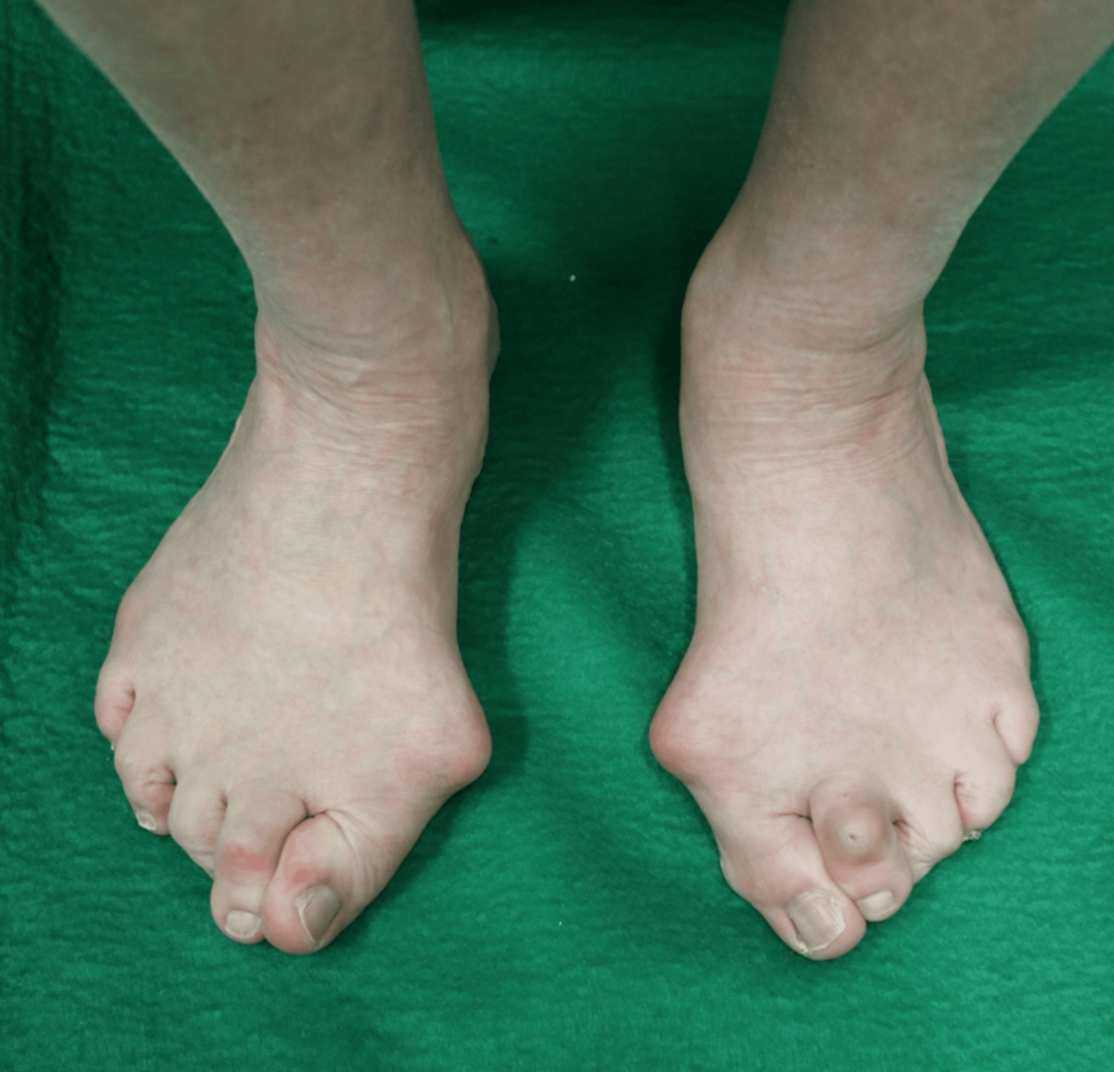
Clinical Images of preoperative hallux valgus Pain in the bunion area was mild, but the hallux was pressing on the second ray. Callosities were seen on the plantar side of the second metatarsal head on both sides, and the dorsal side of the left second proximal interphalangeal (PIP) joint.

She had advanced hallux valgus on both sides, with flat feet and metatarsus latus, but only mild pain in the bunion area. The hallux was pressing on the second ray on both sides. Callosities were seen on the plantar side of the second metatarsal head on both sides and the dorsal side of the left second proximal interphalangeal joint (Figure 1).

**Figure 2:**
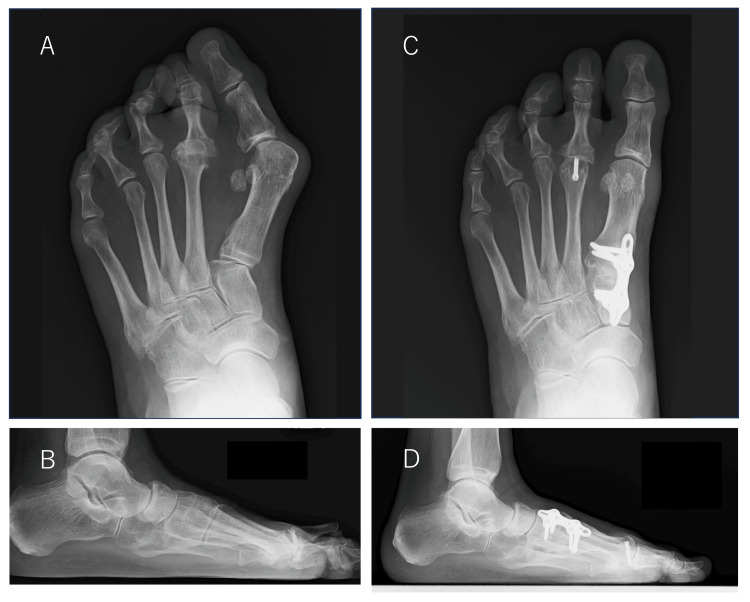
Weightbearing plain radiographs of the left foot Preoperative plain radiographs (A, front; B, side) show that the hallux valgus angle was 51°, the first-second intermetatarsal (M1M2) angle was 24°, the talus-first metatarsal angle was -8°, and the round sign was positive. Postoperative plain radiographs (C, front; D, side) show that the hallux valgus angle was 17°, the M1M2 angle was 11°, the talus-first metatarsal angle was 8°, and the round sign was negative. The correction position was good.

**Figure 3:**
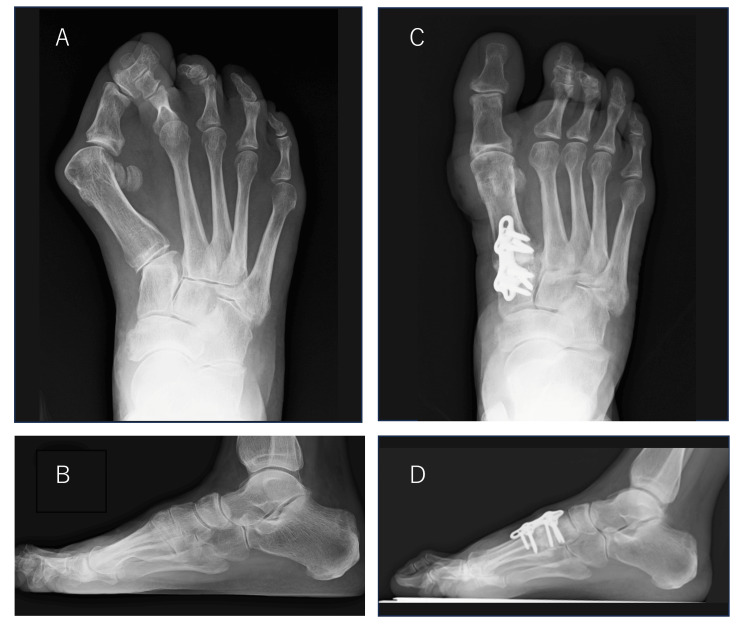
Weightbearing plain radiographs of the right foot Preoperative plain radiographs (A, front; B, side) show that the hallux valgus angle was 55°, the first-second intermetatarsal (M1M2) angle was 26°, the talus-first metatarsal angle was -10°, and the round sign was positive. Postoperative plain radiographs (C, front; D, side) show that the hallux valgus angle was 9°, the M1M2 angle was 8°, the talus-first metatarsal angle was 9°, and the round sign was negative. The correction position was good.

Plain radiographs showed that the hallux valgus angle was 55° on the right and 51° on the left, the M1M2 angle was 26° on the right and 24° on the left, the sesamoid bone deviation was 7° on the right and 7° on the left, according to the Hardy classification [7], and the round sign was positive in both feet (Figures 2A, 2B, 3A, 3B) [8].

The patient was diagnosed with severe bilateral hallux valgus. Conservative treatment with insoles was ineffective, and the pain interfered with her day-to-day activities; therefore, surgery was deemed necessary.

First, we performed a modified Lapidus procedure and Weil osteotomy to reduce the dislocated metatarsophalangeal (MTP) joint in the second ray on the left foot. The caput transversum and obliquum of the adductor hallucis were separated on the lateral side of the MTP joint. Next, a longitudinal incision was made on the lateral side of the MTP joint capsule. It was confirmed that the adduction contracture of the hallux was released, and the hallux valgus was corrected. A modified Lapidus procedure was then performed, the first TMT joint was deployed, and a subchondral osteotomy was performed. The cartilage was first resected with a curette, after which the subchondral bone was minimally resected with a bur to preserve the shape of the articular surface. The first TMT joint was then fixed with a plate by pulling the first metatarsal toward the second metatarsal while keeping the joint in plantar flexion and correcting the rotation. Immediate postoperative measurements for the left foot showed a hallux valgus angle of 17°, an M1M2 angle of 11°, a talus-first metatarsal angle of 8°, and a negative round sign, with a good correction position (Figures 2C, 2D). One year after surgery, the hallux valgus angle was 5°, the M1M2 angle was 7°, and the talus-first metatarsal angle was 4°. Her Japanese Society for Surgery of the Foot (JSSF) standard rating scale score increased from 54 points preoperatively to 90 points after surgery [9,10]. The SAFE-Q (Self-Administered Foot Evaluation Questionnaire) subscale scores ranged from 65 to 99 points for pain-related scores, 75 to 93 points for physical function and daily living status, 45 to 100 points for social functioning, 50 to 91 points for shoes-related scores, and 60 to 95 points for overall health [11].

Two months later, the same modified Lapidus procedure was performed on the right side. A natural medial longitudinal arch was created on both sides, and the hallux valgus was corrected. Preoperative and immediate postoperative measurements for the right foot showed that the hallux valgus angle decreased from 55° to 9°, the M1M2 angle decreased from 26° to 8°, the talus-first metatarsal angle increased from -10° to 9°, and the round sign changed from positive to negative, indicating good correction (Figures 3C, 3D).

**Figure 4:**
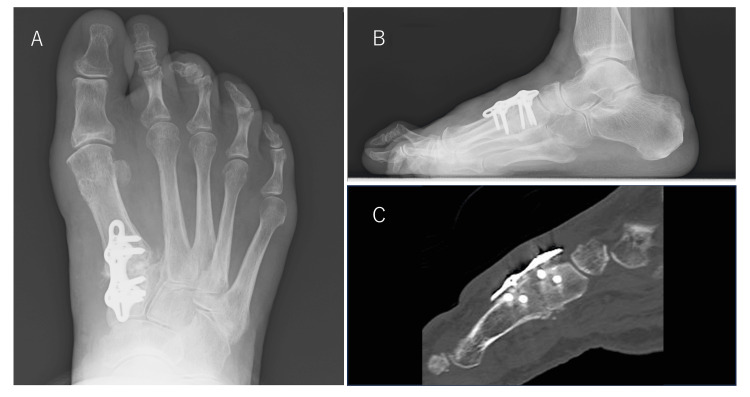
Weight-bearing plain radiographs of the right foot taken at two months postoperatively Plain radiographs (A, front; B, side) show a loss of correction of the hallux valgus angle of 24° and the first-second intermetatarsal (M1M2) angle of 14°. The round sign remained negative, but there was a significant loss of correction in the talus-first metatarsal angle, -12°. A computed tomography (CT) image (C, sagittal) shows bone translucency around the proximal screw and a bone gap at the first tarsometatarsal (TMT) joint.

Heel-walking was permitted from the first postoperative day, and load-walking was started under an orthosis that avoided forefoot loading two weeks later. At two months postoperatively, the patient was pain-free, but her hallux valgus had recurred on the right. Plain radiographs showed loss of correction of the hallux valgus angle, which had increased to 24°, and of the M1M2 angle, which had increased to 14°. The round sign remained negative, but there was a significant loss of correction in the talus-first metatarsal angle, which was -12° (Figures 4A, 4B).

**Figure 5:**
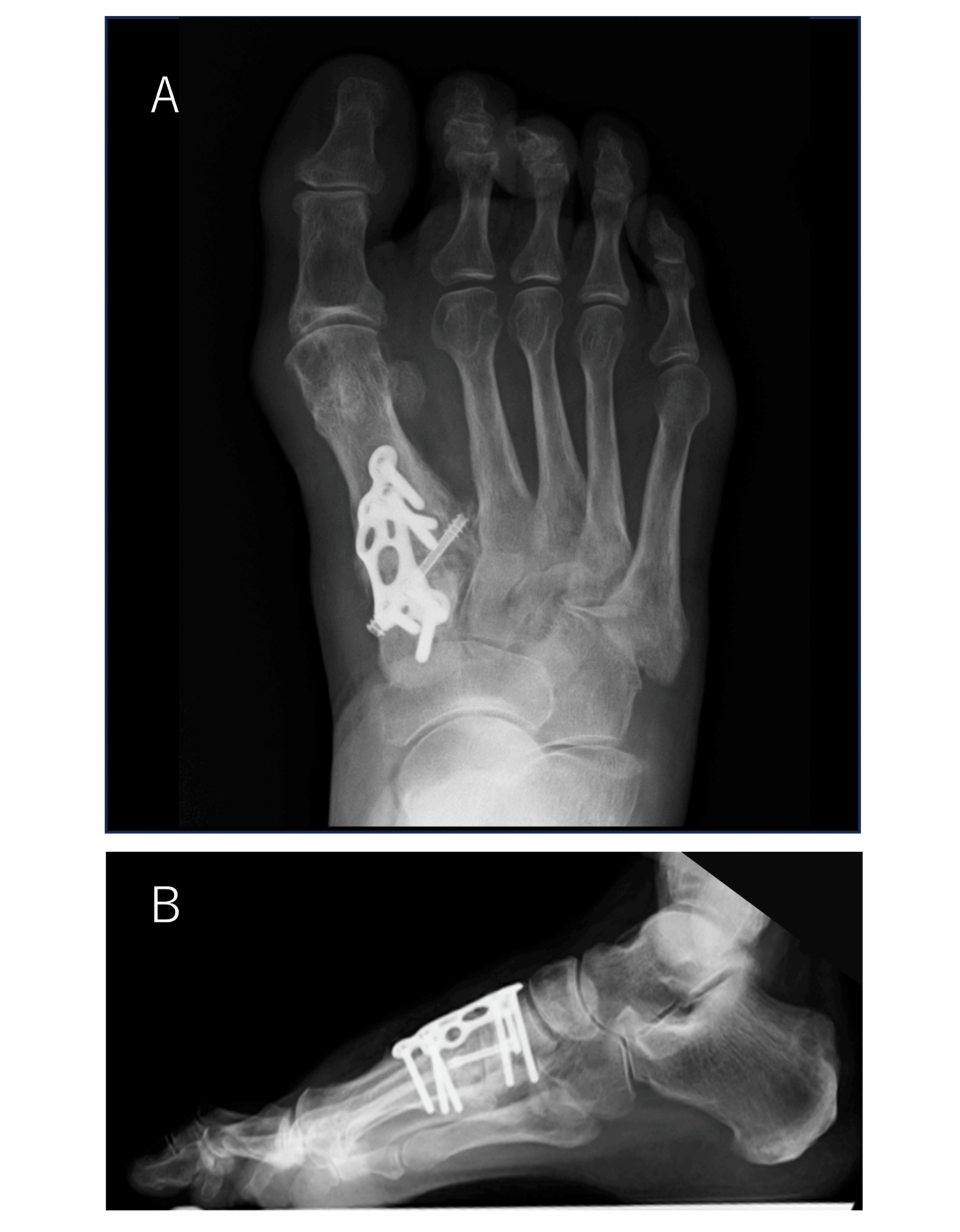
Re-postoperative loading weight-bearing plain radiographs of the right foot Plain radiographs (A, front; B, side) show that the hallux valgus angle was 8°, the first-second intermetatarsal (M1M2) angle was 10°, the talus-first metatarsal angle was 4°, and the round sign was negative.

A computed tomography (CT) image showed bone translucency around the proximal screw and a bone gap at the first TMT joint (Figure 4C). We considered the loss of correction to have been caused by displacement of the bone junction before union and by dorsiflexion and abduction at the first TMT joint. The patient underwent revision surgery two months after the first surgery. After the removal of the previous plate, the first TMT joint was resurfaced and corrected again, with a focus on correction in the sagittal plane. The joint was temporarily fixed with a K-wire. After grafting iliac bone into the gap, repeat fixation was performed using a plate with a shape different from that used in the initial surgery. A screw was added to penetrate the first TMT joint. Immediately after surgery, the hallux valgus angle was 8°, the M1M2 angle was 10°, the talus-first metatarsal angle was 4°, and the round sign was negative (Figures 5A, 5B).

**Figure 6:**
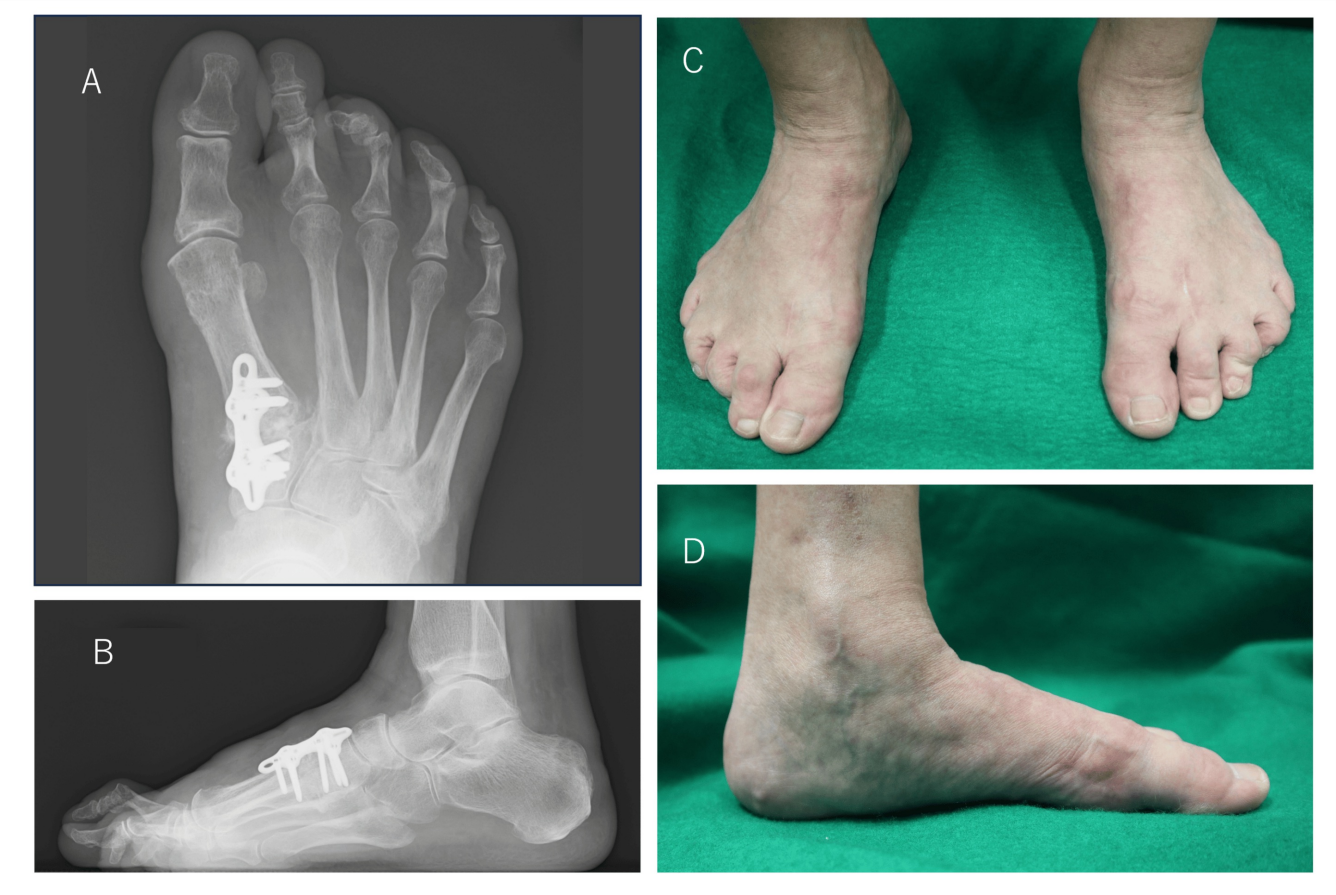
Weight-bearing plain radiographs of the right foot taken one year postoperatively Plain radiographs (A, front; B, side) show that the hallux valgus angle was 14°, the first-second intermetatarsal (M1M2) angle was 10°, and the talus-first metatarsal angle was 3°. Clinical images of postoperative hallux valgus (C, front; D, side) show a good correction position.

Postoperative treatment was the same as that after the previous surgery. One year after surgery, the hallux valgus angle in the right foot was 14°, the M1M2 angle was 10°, and the talus-first metatarsal angle was 3°, with a good correction position maintained and no pain (Figures 6A-6D).

The patient’s JSSF scale score increased from 67 points preoperatively to 100 points after surgery [9,10]. The postoperative SAFE-Q subscale scores were 77 to 91 points for pain, 93 to 100 points for physical function and ability to perform activities of daily living, 100 points for social functioning, 100 points for shoe-related problems, and 100 to 95 points for overall health [11].

## Discussion

Hallux valgus is not only “valgus of the hallux” but also includes varus of the first metatarsal, flat foot, metatarsus latus, laxity of the medial support mechanism, pronation of the hallux, and deviation of the sesamoid bone - all of which interact to produce a complex pathology. We believe that hypermobility of the first TMT joint plays a central role in this condition.

**Figure 7:**
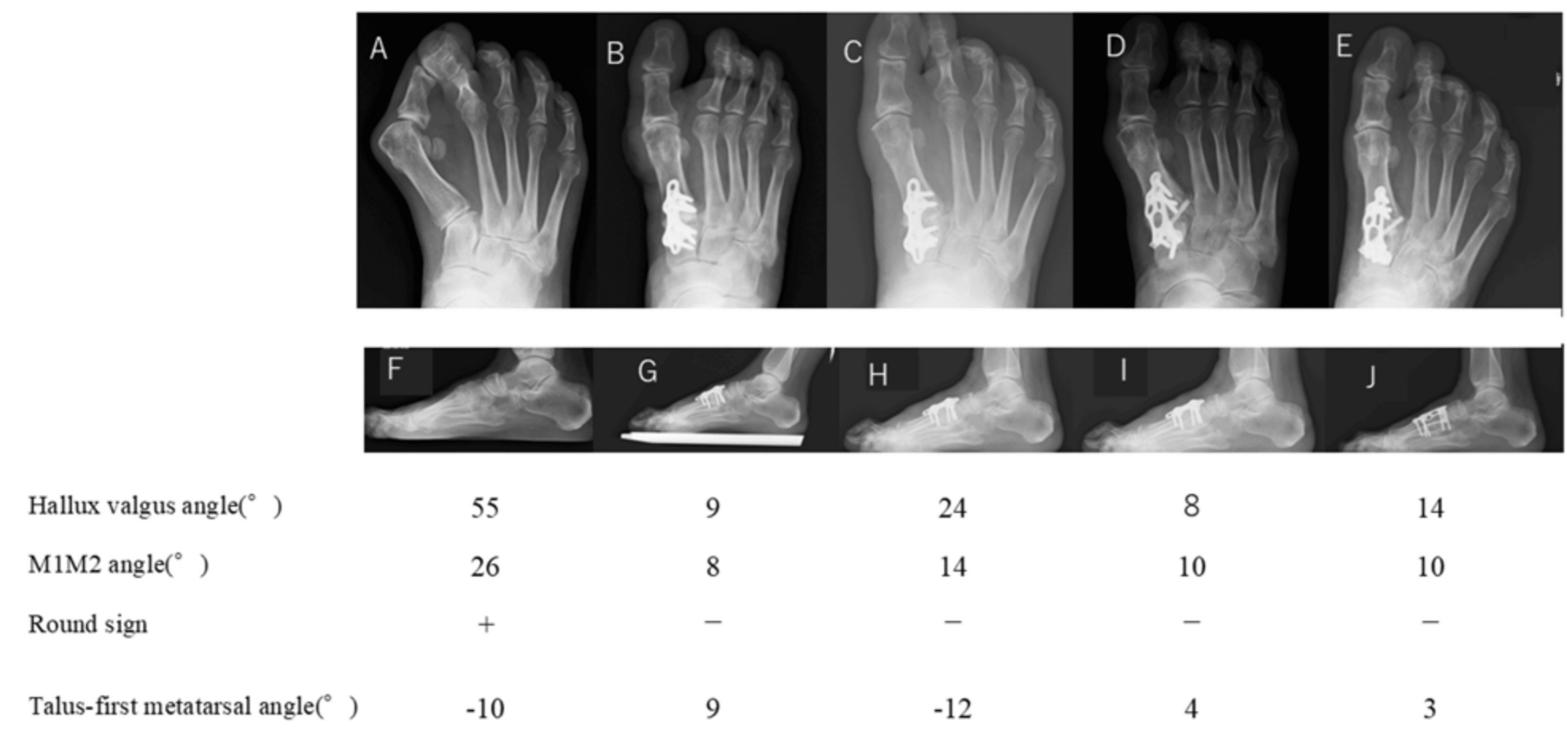
(A)-(J) Changes in alignment over time shown in weight-bearing plain radiographs of the right foot When hallux valgus recurred in the present case, the round sign remained negative, and there was little rotation of the first metatarsal head or distal phalanx. However, the deformity recurred due to significant loss of the talus-first metatarsal angle (21°) and slight opening of the first-second intermetatarsal (M1M2) angle (6°) (B, C, G, H).

In a previous weight-bearing CT study with loading equivalent to body weight, we observed hypermobility of the first TMT joint in patients with hallux valgus. In patients with hallux valgus, the talus-first metatarsal angle was significantly smaller than in those with normal feet, and the first metatarsal was in dorsiflexion and varus when loaded [12], meaning that the position of the first metatarsal head is displaced medially and dorsally by loading. The medial longitudinal and transverse arches are simultaneously reduced. Tension in the tendons and plantar fascia attached to the hallux valgus increases, but because the first metatarsal is varus, the proximal phalanx is pulled in the valgus direction, accompanied by further supination. Reconstruction of the medial longitudinal arch is necessary when correcting hallux valgus with advanced three-dimensional deformity because the deformity will recur if these soft tissue tensions are not released. Indeed, when hallux valgus recurred in the present case, the round sign remained negative, and there was little rotation of the first metatarsal head or distal phalanx; however, the deformity recurred as a result of significant loss of the talus-first metatarsal angle (21°) and slight opening of the M1M2 (6°) (Figures 7A-7J).

This suggests that the flattening of the medial longitudinal arch leads to the recurrence of the deformity, even if the correction of the other components of hallux valgus is maintained.

We considered that a modified Lapidus procedure was suitable for our patient, who had a very large hallux valgus angle and significant hypermobility of the first TMT joint. Good correction was achieved after the initial surgery in both feet. However, early postoperative loss of correction occurred in the right foot, which had undergone surgery later than the left foot. The recurrence rate after a modified Lapidus procedure has been reported to range from 3.6% to 12% [13]. Factors that may cause recurrence include insufficient fixation force due to problems with the material or surgical technique [14,15], bone fragility, poor patient compliance with loading restrictions, and early loading within two weeks [13,16]. Biomechanically, the combination of a plate and screws is advantageous [17-19], but it was not performed in the initial surgery on the right foot in this case because there had not been any problems with surgery on the left foot. However, bone fragility due to rheumatoid arthritis should have been considered. The short interval of two months after the contralateral surgery, which had had a good postoperative course, may have tempted the patient to increase loading on the forefoot too soon in the early postoperative period. There has been some debate about how to deal with recurrence after a modified Lapidus procedure. In our case, we attributed the recurrence of hallux valgus deformity to increased forefoot loading before bone union was achieved, resulting in a loss of correction of the talus-first metatarsal angle and M1M2, which again increased the extra-articular soft tissue tension. To release the soft tissue tension, we reoperated with a focus on correction of the sagittal alignment and were able to re-correct the deformity. We also used a combination of a plate and screws and provided the patient with strict instructions regarding careful loading and gait restrictions. The hallux valgus did not recur, and a well-corrected position was maintained.

## Conclusions

We report a case of revision surgery for a foot with loss of correction after a modified Lapidus procedure. Even with a modified Lapidus procedure, early loading when walking can cause a significant loss of correction.

In this case, dorsiflexion of the first metatarsal was greater than the opening M1M2 angle, with less rotation of the first metatarsal head. The main cause of the loss of correction was dislocation at the first TMT joint caused by dorsiflexion, which could be corrected to achieve a good position again.
